# Clinical Re-Audit of Assessment and Recording of Venous Thromboembolism (VTE) in Patients With Confirmed COVID-19 in Forensic SIS (Secure Inpatient Services)- 2020/2021

**DOI:** 10.1192/bjo.2022.329

**Published:** 2022-06-20

**Authors:** Bhavana Piparsania, Santosh Kumar, Raminderjit Kaur

**Affiliations:** Tees, Esk and Wear Valley NHS Foundation Trust, Middlesbrough, United Kingdom

## Abstract

**Aims:**

1. To assess quality of VTE risk assessment and recording; particularly to look at the impact of COVID-19 on VTE (Venous Thrombo-embolism). 2. To ensure VTE criteria have been adhered to from Tees, Esk and Wear Valleys NHS foundation trust 2019 ” Risk assessment for Venous Thromboembolism (VTE) (Ref: CLIN-0085-v 1.2)Specifically, due to additional risks posed by COVID-19 on increase in risk of VTE.

**Methods:**

The Audit was conducted in Secure inpatient service in Teesside, Roseberry Park Hospital, TEWV NHS trust audit team.

This was done on the back of a scheduled Quality improvement reaudit of VTE risk assessment and recording review in Forensic SIS.

**Results:**

One of the main results was that VTE risk assessment and recording, post finding of a COVID-19 positive result was less than 100% in the records we checked. Other results are included in the poster.

**Conclusion:**

The main conclusion is the need for increased vigilance in assessment and recording of (and any actions thereof) in the VTE risks, particularly in those with COVID-19 positive tests.

We propose that this increased vigilance will enhance patient safety and deliver effective and timely care. We highlight some challenges of conducting an Audit and how to embed results and improve practices in a timely manner. This describes how we did both- acted on results and followed process; rather than just one or the other!

